# Prediction of water distribution uniformity of sprinkler irrigation system based on machine learning algorithms

**DOI:** 10.1038/s41598-023-47688-3

**Published:** 2023-11-28

**Authors:** Khadiga T. Elhussiny, Ahmed M. Hassan, Ahmed Abu Habssa, Ali Mokhtar

**Affiliations:** 1 Department of Agricultural Engineering, Faculty of Agriculture, Cairo University, Giza, 12613 Egypt; 2 Department of Mechanical Power, Mataria Faculty of Engineering, Helwan University, Helwan, Egypt

**Keywords:** Applied mathematics, Computational science

## Abstract

The coefficients of uniformity Christiansen's uniformity coefficient (CU) and distribution uniformity (DU) are an important parameter for designing irrigation systems, and are an accurate measure for water lose. In this study, three machine learning algorithms Random forest (RF), extreme gradient boosting (XGB) and random forest-extreme gradient boosting (XGB-RF) were developed to predict the water distribution uniformity based on operating pressure, heights of sprinkler, discharge, nozzle diameter, wind speed, humidity, highest and lowest temperature for three different impact sprinklers (KA-4, FOX and 2520) for square and triangular system layout based on four scenarios (input combinations). The main findings were; the highest CU value was 86.7% in the square system of 2520 sprinkler under 200 kPa, 0.5 m height and 0.855 m^3^/h (Nozzle 2.5 mm). Meanwhile, in the triangular system, it was 87.3% under the same pressure and discharge and 1 m height. For applied machine learning, the highest values of R^2^ were 0.796, 0.825 and 0.929 in RF, XGB and XGB-RF respectively in the first scenario for CU. Moreover, for the DU, the highest values of R^2^ were 0.701, 0.479 and 0.826 in RF, XGB and XGB-RF respectively in the first scenario. The obtained results revealed that the sprinkler height had the lowest impact on modeling of the water distribution uniformity.

## Introduction

Water scarcity is a grave challenge for cultivation, particularly in arid and semiarid regions, whereas, the development of novel water supplies requires high costs^1^. The efficient use of water and energy in agriculture is becoming more important due to decreasing water resources and increasing cost of energy over the world^2^. In fact, agriculture is responsible for nearly 70% of all fresh water withdrawn in the world, most of it through irrigation (FAO 2015)^3^. Thus, increasing the distribution uniformity of water not only leads to the perfect use of the available water resources, however, it also increases the efficiency of water management^1^. Many studies have informed that the heterogeneity in water distribution has a negative impacts on the efficiency of water management^1^. Furthermore, the pressurized irrigation systems such as drip and sprinkler irrigation have become more efficient and feasible to apply to increase the water use efficiency^4,5^.

The coefficient of uniformity is often considered one of the mostly important criteria for water distribution efficiency in the design and management of sprinkler irrigation systems^6,7^. The sprinkler irrigation distribution patterns have been characteristic of diverse statistical uniformity coefficients^8^ and several coefficients of uniformity have been applied over the last years^9^. Quantitatively, can be apply various criteria to estimate the uniformity of water distribution in sprinkler irrigation systems, the Christiansen’s uniformity coefficient is widely applied in irrigation systems, among others^10^. Christiansen's uniformity coefficient^11^ was used for the first time to calculate the uniformity coefficient for a sprinkler system on the universal level^8,12^.

When irrigation is applied by the sprinkler method, water is distributed over the irrigated area by spraying it through the air. Nozzles may be rotated to cover circular or part circle land areas, or they may be fixed and equipped with deflectors that break up the water stream and deflect it onto the area to be irrigated. High uniformity of water application is achieved by overlapping the spray patterns from adjacent sprinklers^13^.

Distribution uniformity (DU) is the percentage of average application amount received in the least-watered quarter of the field, it gives an indication of the magnitude of the distribution problem^14^.

Distribution uniformity (DU) or pattern efficiency. This method sort all data point in the overlap area and tanks then from low to high with mean value for lowest 25% (low quarter) divided by mean value for the entire area. However, this method does not take into account the location of water value or any benefit, which might be divided from water value, immediately adjacent to the low values^15^.

The distribution efficiency can be obtained by the distribution uniformity, which consists in a measurable capacity of an irrigation system to apply an equal amount of water in the irrigated perimeter^13,16^. The distribution uniformity for different irrigation types is also influenced by different factors, related to each technique of irrigation^17^. Particularly for sprinkler systems, the uniformity is related not only to its mechanical aspects (flow rate, operating pressure, spacing, nozzle diameter, etc.), but also to meteorological conditions, especially wind direction and speed^18,19^. Its estimate is frequently evaluated based on the uniformity coefficients.

On the other hand, several parameters can affect the uniformity of water distribution on a farm level such as climate and hydraulic parameters. These criteria involve sprinkler construction, angle of spray, diameter of nozzle, operating pressure, height of raiser, spacing between sprinklers and average wind speed values^20,21^. However, the most important factors affected in uniformity of irrigation are the operating pressure and the diameter of the nozzle^2^.

On the other hand, some computer-based models have been already utilized for estimation of water distribution uniformity, despite they have not comprised comprehensive scopes.

These models can be categorized split on three models, namely ballistic, semi empirical, and statistical models^22^. Nevertheless, these models take into account some presupposition, ignore some efficacious variants, and demand a much of data as atmospheric and hydraulic. For this reason, it is difficult to apply these models to the design, management, and use of irrigation systems^23,24^. For that reason, appropriate methods need to be used to estimate both of CU and DU values with acceptable accuracy, in less time, and at lower computational cost. Due to the aforementioned methods, artificial intelligence (AI) techniques can be used to evaluate the results of CU and DU with appropriate execution fineness.

Over the last few years, many studies have mentioned that the prosperous applications of AI techniques in various scope of agricultural and water engineering issues^25–27^. Application of probabilistic linear regression (PLR), random forest (RF), and cross-correlation function to study the effective rate of climatic parameters on the potential evapotranspiration of barley through the growing season was reported by^28^. The support vector regression (SVR) with hybrid nature-based algorithms was applied to model soil cation exchange capacity^29^. Various applications of these techniques were applied in pan evaporation^30^, crop water requirement^31–33^, drought indices^34,35^, friction coefficient of irrigation pipes^36^, micro irrigation filters^37^, simulation of qualitative parameters for surface water/groundwater^38–41^, dew point temperature^42^ and soil parameters in irrigated land^43,44^. Therefore, the main objectives of the present study were to investigate the applying RF, XGB and XGB-RF models for predicting the water distribution uniformity fixed sprinkler solid-set systems under the arid region climate. The major objectives for that research were to; (1) Evaluate the water distribution uniformity based on CU and DU for the fixed sprinkler solid-set systems, (2) Investigate the possibility of providing a unique single and hybrid machine learning model to predict the CU and DU and (3) Select the best scenario from the input parameters to successfully predict the CU and DU of the sprinklers irrigation systems. This research is critical in determining the best approach (optimal model and input variables) that could be used as a simple, rapid, and inexpensive approach for timely and reliable water distribution uniformity of sprinkler irrigation system in Egypt. To the best of our knowledge, the applied approaches are still poorly investigated for water distribution uniformity, especially those based on different climate inputs and hydraulic parameters. So, the novel contribution of this work is to develop and compare the results from the single and hybrid machine learning algorithms under the arid regions.

## Materials and methods

### Study area

The field experiments were performed at the Agricultural Engineering Department of Faculty of Agriculture, Cairo University, Egypt (latitude 30.0861 N, longitude 31.2122 E, and altitude 19 m above sea level). The soil of the experimental area is classified as sandy clay loam. Irrigation water for the sprinkler system was obtained from a deep well. The meteorological variables (maximum and minimum air temperature, relative humidity and the wind speed) were obtained from a near meteorological station as an hourly time scale. The maximum temperature during the experiments period (from 3 August 2022 to 5 September 2022) ranged from 85 to 94.3 °F (26 to 36.2 °C), wind speed ranged from 0 to 4 m/s and the air humidity of the air ranges from 37 to 82%.

### Field experiments

**Figure 1:**
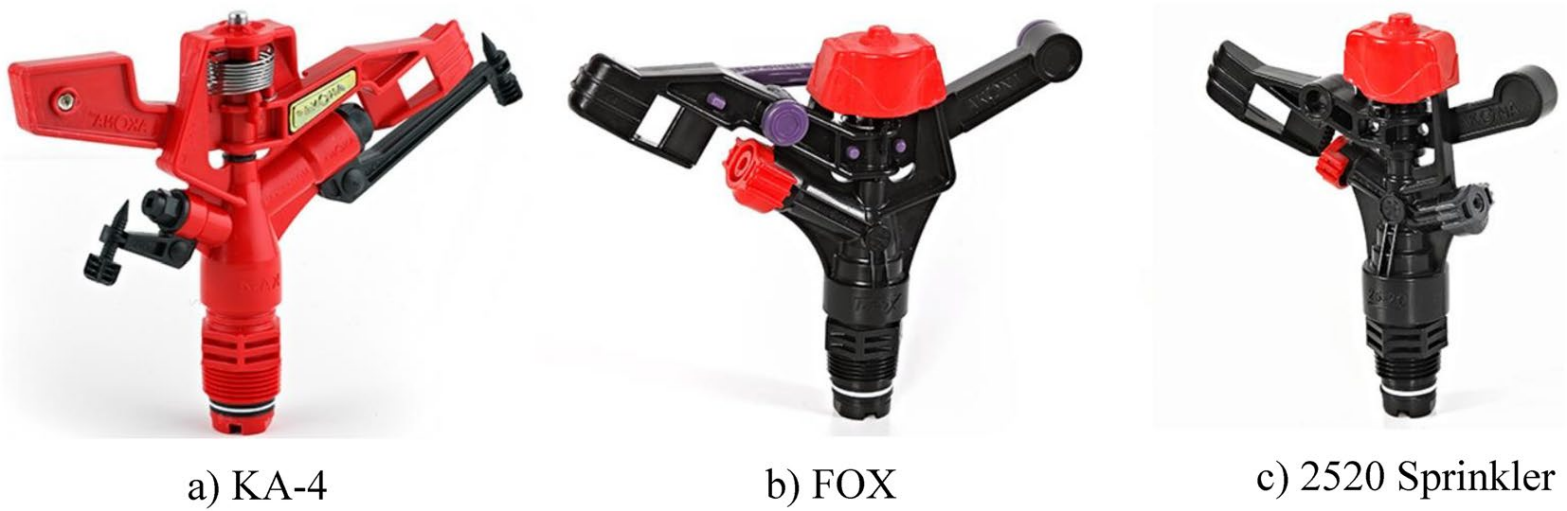
The three impact sprinklers applied.

**Figure 2:**
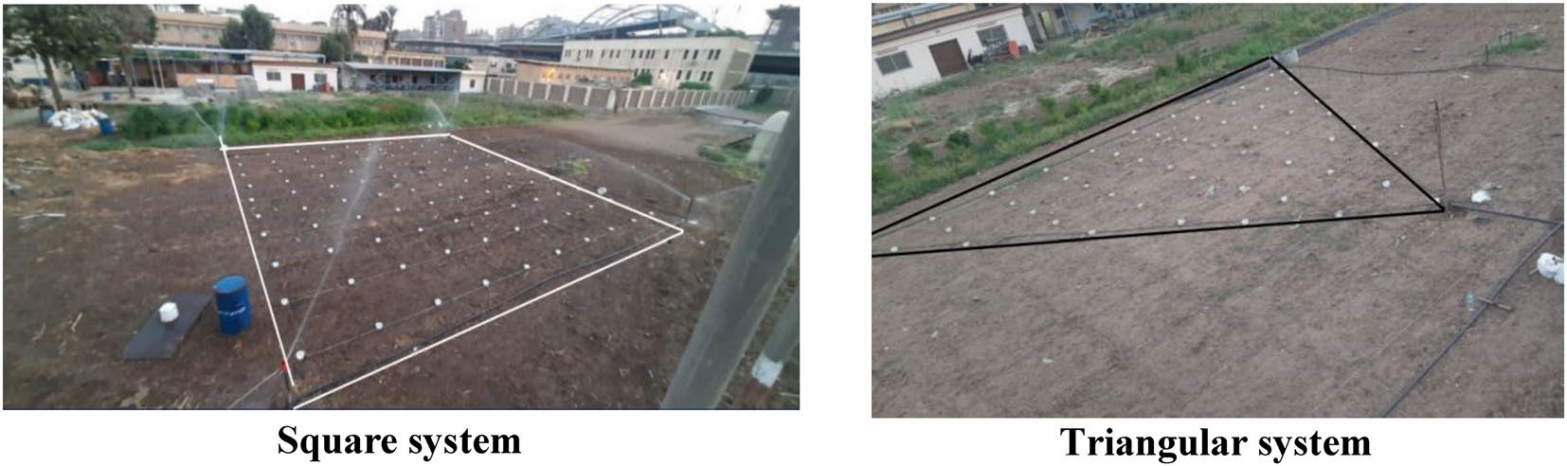
Sprinkler systems layouts.

Three impact sprinklers were used for measuring the CU and DU values based on hydraulics parameters (operating pressure, heights of sprinkler, and nozzle diameter) (Fig. 1). The operating pressures were set as 150, 200 and 250 kPa, the height of sprinkler riser was 0.5, 1 and 1.5 m and the nozzle diameter was 2.5, 3.8 and 4.2 mm (Table 1). The experimental set-up included a deep well, submersible pump, main and sub main pipe, pressure gauge, sprinklers and catch cans to collect water from the sprinklers during the experiments. Two systems layouts were used for the experiments the square and triangular system (Fig. 2). The total number of experiments were 54 experiments, 27 for the square system (3 operating pressure × 3 heights × 3 nozzles diameter) and 27 for the triangular system. Adjusting the required pressure for each experiment is applied before starting each experiment.

**Table 1:** Characteristics of the sprinklers used.

Sprinklers type	Number of nozzles	Nozzle diameters (mm)	Throw range (m)
sprinkler KA-4	2	4.2 and 4.2	15–22
sprinkler FOX	2	3.8 and 1.8	14.9–20.3
sprinkler 2520	2	2.5 and 1.8	9–11

### Water distribution uniformity

Christiansen's distribution uniformity of water as a reference technique counselled by the last researches was evaluated through the Christiansen's uniformity coefficient^24^. The coefficient of uniformity developed by^11^:

1 $$CU = 100\left( {1 - \frac{{\sum {\left| {z - m} \right|} }}{\sum z }} \right)$$

where CU coefficient of uniformity developed by Christiansen (%), z individual depth of catch observations from uniformity test (mm). X = I z − m I = absolute deviation of the individual observations from the mean (mm). m = (∑z)/n = mean depth of observations (mm). n = numeral of observations.

The second widely used criterion for estimating sprinkler irrigation uniformity is uniformity of distribution as it is a useful term for inclusion a numerical value on the uniformity of application of agricultural irrigation systems, DU^45^. It was measured by testing catch cans after irrigation system has been installed to verify distribution uniformity. For this test, the cans were distributed evenly in the area under the study. The distribution uniformity of low quarter, the calculation was performed according to Burt et al.^46^.

2 $$DU_{lq} = \frac{{d_{lq} }}{{d_{avg} }}$$

where DU_lq_ = distribution uniformity low quarter. D_lq_ = the lowest quarter depth (lowest 25% of the observed depths). D_avg_ = the average depth of the total sprinklers (cans).

### Machine learning models applied

#### Extreme gradient boosting (XGB)

The Extreme Gradient Boosting (XGB) algorithm suggested by^47^ it is a recent improvement of the regression trees based gradient boosting machine. The algorithm is on the basis of the idea of “boosting”, which combines all the predictions of a set of “weak” learners to develop a “strong” learner through additive training strategies. XGB model is designed to avoid overfitting while still optimizing computing resources. This is done by simplifying the target functions in such a way that they can combine predictive and regularization terms while also retaining a high computational speed. During the training process of XGB, parallel simulations are also performed automatically for the functions. In the XGB additive learning procedure, the learner is fitted initially with the entire input space, and then a second model with the residuals is fitted to overcome a weak learner's disadvantages. This fit is performed until the stop criteria has been met. The final forecast of the model is computed according to the number of predictions of each learner. The general function is presented as follows:

3 $$f_{i}^{(t)} = \sum\nolimits_{k = 1}^{1} {f_{k} (x_{{_{i} }} )} = f_{i}^{(t - 1)} + f_{t} (x_{i} )$$

where $f_{i}^{(t)}$ and $f_{i}^{(t - 1)}$ represent the forecasts in steps t, $f_{t} (x_{i} )$ is the learner step by step, and (*t* − 1), and *x*_*i*_ is the input variable. Chen and Guestrin^47^ have extensive details and calculations for the XGB algorithm.

To select the best model. XGB was applied by using a learning rate = [0.5, 0.1, 0.05] the best one is 0.5, n_estimators = [50, 100, 200, 400, 600, 500, 1000] the best one is 500 and max depth = [1, 2, 5, 10, 12, 15] the best one is 1. For CU, the best learning rate for the first is 1, the second and the third scenarios is 0.5 and for the fourth scenario is 0.05. However, the best n_estimators one for the first scenario is 500, for the second scenario is 100, for the third scenario is 50 and for the fourth scenario is 400. On the other hand, the best max_depth for the first, the third and the fourth scenarios is 1 and for the second scenario is 5, the best Score for the first scenario is 0.761, for the second scenario is − 0.577, for the third scenario is 0.771 and for the fourth scenario is 0.738.

For DU, the best learning rate for the first is 0.1, the second, the third and the fourth scenarios is 0.05. However, the best n_estimators one for the first, the third and the fourth scenarios is 200 and for the second scenario is 100. On the other hand, the best max_depth for the four scenarios is 1, the best Score for the first scenario is 0.468, for the second scenario is − 0.522, for the third and for the fourth scenario is 0.671.

#### Random forest (RF)

The RF model, devised by^48^, is based on a group of verification trees with controlled variance; the RF model is widely used for inverse transformations and classification problems. Random forest is designed to form an ensemble of weak unbiased classifiers which combine their results during the final classification of each object. Individual classifiers are built as classification trees. Each tree is constructed using different bootstrap sample of the training set. Each bootstrap sample is a result of drawing with replacement the same number of objects as in the original training set. As a result, roughly 1/3 of objects is not used for building a tree and instead is used for performing an out of bag (OOB) error estimate, and for importance measurement. At the each step of the tree construction a different subset of attributes is randomly selected^49^.

The RF classification algorithm is relatively quick, can usually be run without tuning of parameters and it gives a numerical estimate of the feature importance. It is an ensemble method in which classification is performed by voting of multiple unbiased weak classifiers decision trees. These trees are independently developed on different bagging samples of the training set. The importance measure of an attribute is obtained as the loss of accuracy of classification caused by the random permutation of attribute values between objects. It is computed separately for all trees in the forest which use a given attribute for classification. Then the average and standard deviation of the accuracy loss are computed. Alternatively, the Z score computed by dividing the average loss by its standard deviation can be used as the importance measure. Unfortunately, the Z score is not directly related to the statistical significance of the feature importance returned by the random forest algorithm, since its distribution is not N (0, 1)^50^. RF was applied by using a n_estimators = [50, 100, 200, 400, 600, 500, 1000] and max_depth = [1, 2, 5, 10, 12, 15].

For CU, the best n_estimators one for the first and the second scenarios is 50, for the third scenario is 500 and for the fourth scenario is 1000. On the other hand, the best max_depth for the first, the third and the fourth scenarios is 2 and for the second scenario is 10, the best Score for the first scenario is 0.761, for the second scenario is -0.185, for the third scenario is 0.771 and for the fourth scenario is 0.778.

On contrast, For DU, the best n_estimators one for the first scenario, the second and the third scenarios is 50 and for the fourth scenario is 400. On the other hand, the best max_depth for the four scenarios is 2, the best Score for the first scenario is 0.668, for the second scenario is 0.674, for the third scenario is 0.708 and for the fourth scenario is 0.692.

#### Hybrid XGB and RF

The hybridization between the RF and XGB aimed to improve the performance of single models. Every single model was described in the preceding sections. The hybrid model of XGB-RF is described in Fig. 5, it shows that the initial database is divided into training (70%) and test (30%) sets. Hybrid XGB and RF was applied by using a n_estimators = [50, 100, 200, 400, 600, 500, 1000] and max_depth = [1, 2, 5, 10, 12, 15].

For CU, the best learning rate for the first, the second and the third scenarios is 0.05 and for the fourth scenario is 0.1. However, the best n_estimators one for the first and the third scenarios is 200, for the second and the fourth scenarios is 100. On the other hand, the best max_depth for the four scenarios is 1, the best Score for the first scenario is 0.772, for the second scenario is − 0.436, for the third scenario is 0.764 and for the fourth scenario is 0.769. For DU, the best learning rate for the four scenarios is 0.05. However, the best n_estimators one for the first scenario and the third scenarios is 100, for the second scenario and the fourth scenarios is 200. On the other hand, the best max_depth for the four scenarios is 1, the best Score for the first scenario is 0.436, for the second scenario is 0.624, for the third scenario is 0.772 and for the fourth scenario is 0.729.

### Performance evaluation

The mean absolute error (MAE) and the root mean square error (RMSE) were used to estimate the applied models^51–53^. Moreover, the range of the SI (scatter index) for the classification of the models is “excellent” if SI < 0.1, “good” if 0.1 < SI < 0.2, “fair” if 0.2 < SI < 0.3, and “poor” if SI > 0.3.

4 $$MAE = \frac{1}{n}\sum\limits_{i = 1}^{n} {\left| {O_{i} - P_{i} } \right|}$$

5 $$RMSE = \sqrt {\frac{1}{n}\sum {\left( {P_{i} - O_{i} } \right)}^{2} }$$

6 $$SI = \frac{RMSE}{{ \overline{O}}}$$

7 $$R^{2} = \left[ {\frac{{\sum\limits_{i = 1}^{n} {(O_{i} - \overline{O})(P_{i} - \overline{P})} }}{{\sqrt {\left( {\sum\limits_{i = 1}^{n} {(O_{i} - \overline{O}_{i} )}^{2} } \right)\left( {\sum\limits_{i = 1}^{n} {(P_{i} - \overline{P})^{2} } } \right)} }}} \right]^{2}$$

where $\overline{O}$ represent the average values of the observed CU and DU, *O*_*i*_ and *P*_*i*_ are the observed and predicted CU and DU, respectively, and *i* is the number of observations. *SD* is the standard deviation of the difference between the measured and estimated value. Further, R^2^ is the coefficient of determination (Table 2).

**Table 2:** Input combinations of the applied models for CU and DU.

Scenario	Input
Sc1	Pressure, discharge, height, Tave, Tmin, Tmax, wind and relative humidity
Sc2	Tave, Tmin, Tmax, wind speed and relative humidity
Sc3	Pressure, discharge and height
Sc4	Pressure and discharge

### Ethics approval and consent to participate

All authors are approval in participation in this research. Authors are agreeing to publish their data prior to submitting their paper to the journal.

## Results and discussions

### Water distribution uniformity

**Figure 3:**
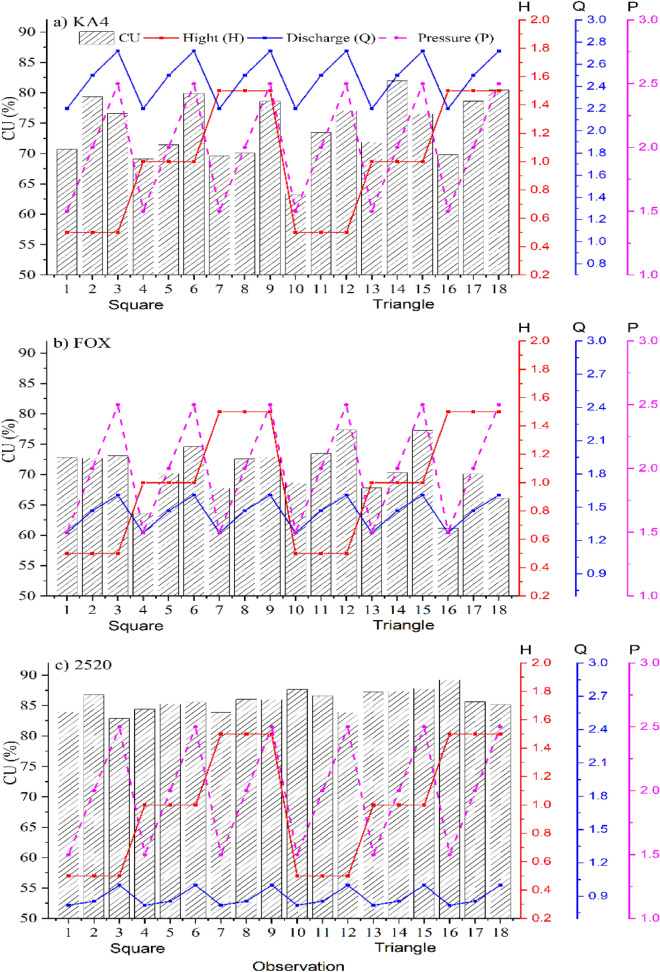
The relationship between the CU of the three selected sprinklers in the square and triangle system and the pressure, discharge and riser height.

These experiments were conducted in the sprinkler irrigation system under three operating conditions and climate parameters to reach the highest possible distribution uniformity as much energy saving as possible. For KA-4 sprinkler, the highest CU value was 79% in the square system under operating pressure of 200 kPa, riser height of 0.5 m and the discharge was 2.5 m^3^/h (Nozzle 4.2 mm) (Fig. 3a). However, it was 81.9% in the triangular system under the same condition but on the 1 m height. Maybe the reason of the low CU at 1 m back to the maximum and minimum temperature reached to 89.7 and 78.5 °F, respectively and the wind speed was 2 m/s (Fig. 5a and Table 3). Furthermore, when the operating pressure 150 kPa was applied, the CU decreased by 10.97% in comparison with 200 kPa in the same height for the square system. Moreover, when applied 150 kPa in comparison with applying 200 kPa in the triangle system, the CU decreased by 27.3%, although, in most cases the CU increased under 250 kPa, except for one experiment in which the value of CU decreased, when using a riser of 1 m height. The reason for this is due to the high maximum temperature which reached 90.1 °F and wind speed was 2 m/s, as the high temperature lead up to the evaporation of some the water collected in the cans during the experiments, accordingly, the wind speed moved some water from cans to others, and thus it affects the uniformity of water distribution. Also, the square system agreed with the triangle system in all cases except when using a height 0.5 m. It is noted that in the square system, when applied 250 kPa, the CU increased by an average rate was 10.65%, while, it was 3.4% in the triangular system, however, the system will consume more energy at 250 kPa, in comparison with operating at 200 kPa, that agree with, the findings of^54^ who mentioned that CU increased with the increased operating pressure until it reached up to 250 kPa. Further, the CU values at 200 kPa in the triangular system, are larger than the square system, so it is preferable to use the triangle system.

**Table 3:** Water distribution uniformity and wind speed (m/s) under the square and triangle sprinklers system.

Observation	Sprinkler type								
	KA4			FOX			2520		
	CU	DU	WS	CU	DU	WS	CU	DU	WS
Square									
1	70.68	62.32	3	72.81	63.73	2	83.92	82.12	1
2	79.35	72.96	2	72.74	68.45	3	86.78	84.67	0
3	76.60	73.24	3	73.09	61.82	1	82.90	77.24	0
4	69.10	68.32	2	63.59	54.21	2	84.43	81.73	3
5	71.42	62.16	2	70.18	59.03	2	85.24	83.91	3
6	79.88	72.59	3	74.59	63.73	2	85.58	82.06	3
7	69.58	66.41	1	67.73	61.76	2	83.86	81.17	4
8	70.09	65.00	1	72.57	62.41	3	86.03	82.49	4
9	78.59	72.50	2	72.84	61.30	3	86.00	82.24	4
Triangle									
1	63.24	4.125	3	8.676	5.055	2	87.64	85.29	1
2	73.47	63.35	3	3.497	0.258	3	86.60	84.03	1
3	77.04	8.286	2	7.367	0.158	3	83.81	79.69	0
4	71.88	2.856	2	7.796	7.895	2	87.26	85.43	1
5	81.98	7.087	3	0.317	8.805	3	7.388	85.45	1
6	76.54	7.246	2	77.26	66.46	3	87.81	84.29	1
7	69.79	9.685	2	1.186	7.204	2	9.228	90.93	3
8	8.637	1.567	3	0.117	9.575	2	85.60	83.01	1
9	0.468	4.367	2	6.036	55.25	2	5.148	82.75	2

**Figure 4:**
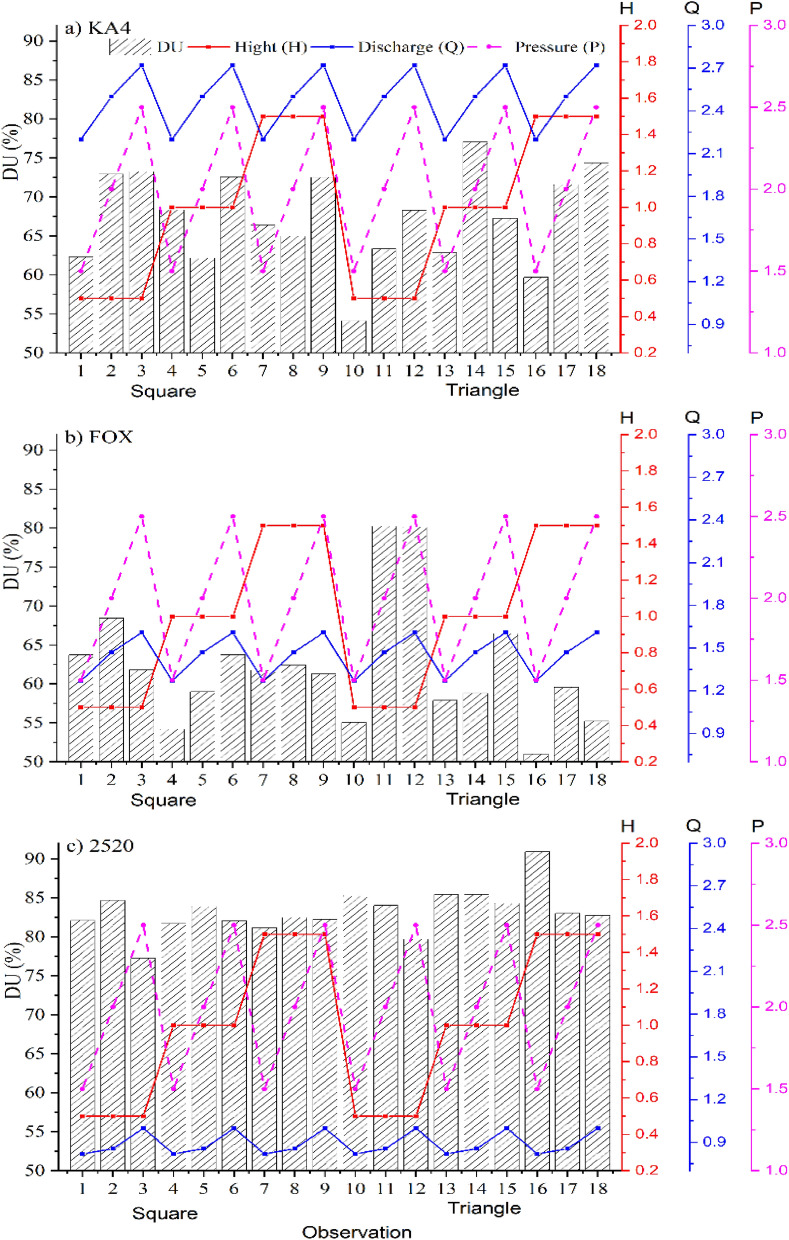
The relationship between the DU of the three selected sprinklers in the square and triangle system and the pressure, discharge and riser height.

**Figure 5:**
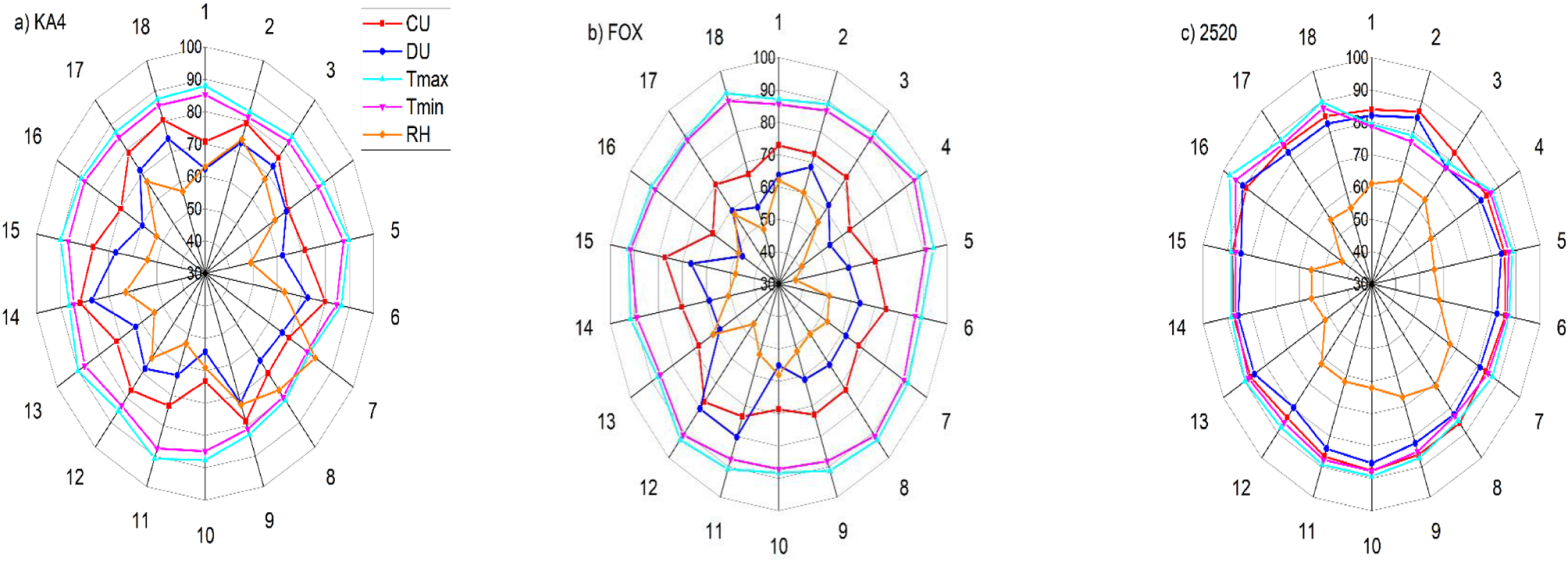
Radar chart of the climate variables with the CU and DU over the square and triangle system.

On the other hand, the highest CU values were 72.7% and 73.4% of the FOX sprinkler for the square and the triangular system, respectively under 200 kPa, 0.5 m height and 1.47 m^3^/h discharge (Fig. 3b), also, the climate variable was closed in the two system during the experiment period (Fig. 5b). In the square system, the temperature was range between (89.1–87 °F), the relative humidity was 60% and wind speed was 3 m/s, and for the triangular system, the temperature was range between (90.8–87.5 °F), the relative humidity and wind speed was 53% and 3 m/s, respectively (Fig. 5b and Table 3). The values of CU when using operating pressures of 200 and 250 kPa were ranged from 70.17 to 86.78% and 72.84 to 86%, respectively, Our results are correspond with those obtained at^24^, which were 73.27 and 77.86% (Fig. 4).

On the other hand, the system consumes more energy when applied the high operating pressure of 250 kPa, where it gives the highest CU values. When applied 250 kPa in comparison with applying 200 kPa, the CU increased in the square system by 2.28%. Moreover, in the triangular system the CU increased in most cases, except for one experiment in which the value of CU decreased by 4%, which may be due to the high wind speed (2 m/s) when applied 250 kPa in comparison with applying 200 kPa. When the operating pressure was 150 kpa, the CU values decreased, while when applying an operating pressure 200 kpa in all experiments, the CU values increased and the system does not take high energy. There is an opposite association between the nozzle height and wind affect and so with drop diameter, the drop diameter under spinner type is smaller than of those for rotating type so we can see that the maximum CU and DU were agreed under 200 kPa operating pressure for both types^55^ (Fig. 5).

Therefore, it is preferable to operate the system by 200 kPa. The lowest value of the CU was 63.5% and 61% in the square and triangular system (Fig. 3b), respectively at 150 kPa, a discharge of 1.27 m^3^/h (Nozzle 3.8 mm) and a riser height of 1 m in the square system but 1.5 m in the triangular system. On contrast, the CU values at 200 kPa in the triangular system are larger than the square system, so it is preferable to use the triangle system.

On the other hand, the highest CU of the 2520 sprinkler under 200 kPa, 0.5 m height and 0.855 m^3^/h (Nozzle 2.5 mm) was 86.7% for the square system, while for the triangular system, it was 87.3% at the same pressure and discharge, and 1 m height (Fig. 3c), furthermore, in the square system, the maximum temperature was 78.8 °F, the minimum was 76.8 °F, the relative humidity was 64% and wind speed was 0 m/s while for the triangular system, the temperature were range between 86.6 and 88.3 °F, the relative humidity was 55% and wind speed was 1 m/s )Fig. 5c and Table 3). Although, the lowest CU value in the two systems at 250 kPa, a riser height 0.5 m, and a discharge of 0.999 m^3^/h, was 82.8% and 83.8% in the square system and the triangular system, respectively (Fig. 3c). The CU decreased in the square system by 2.2% and 1.86%, when applied 150 kPa, in comparison with applying 200 kPa or 250 kPa, respectively. Meanwhile, it does not affect the CU value, therefore it is preferred to apply the 200 kPa in the square system, where, the system does not consume high energy. Therefore, our results are agree with the finding of^2^ who mentioned that the CU was increased when the pressure increased for all the nozzles. In the triangle system, the CU increased by 1.86% and 4% was applied 150 kPa, in compared with applying 200 kPa with a riser height 0.5 or 1.5 m, however, it is not a significant increase does not affect in the CU, while when using 1 m height, the CU was equal at the three pressures, therefore it is preferable to use the triangle system applied by 200 kPa.

On the other hand, the DU increased by 9.6% and 2.3% for the KA-4 sprinkler, when 150 kPa was applied in the square system in comparison with applying 200 kPa through using a 1 m or 1.5 m height (Fig. 4a), which may be due to low the maximum and minimum temperature at 150 kPa in compared to 200 kPa where it ranged from (83.5–85.7 °F) to (87.5, 89.7 °F), respectively for a 1 m height and ranged from (79.1, 79.1 °F) to (79.7, 81.1 °F), respectively for a 1.5 m height. However, the percentage increase is small that it does not affect in the DU value, although, when using 0.5 m, the DU decreased when150 kPa was applied in comparison with 200 kPa, the reason for this is due to the high temperature maximum and minimum (85.5, 88 °F) and (81.4, 83.2 °F), respectively. Which is in agreement with the results of^4^ who conclude that the DU values were low at 0.5 m height and at pressures of 62 and 82 kPa and then increased rapidly with the increasing pressure. On the other hand, the DU decreased when applying 150 kPa in compered to apply 200, 250 kPa in the tringle system, moreover, the difference when was applied 200 kPa and 250 kPa was don’t affect in the DU value. On the other hand, for the FOX sprinkler in the square and tringle system the DU increased when applying 200 kPa in comparison with applying 250 kPa in the most cases (Fig. 4b), which may be due to low the maximum and minimum temperature at 150 kPa, therefore, it is preferred applied by 200 kPa in both systems. In addition to, the DU for 2520 sprinkler in the square system increased when applying 200 kPa in compared to applied 150 kPa, which, may be due to low wind speed in some cases and the other cases may be because of low the maximum and minimum temperature at 200 kPa. Further, when the 200 kPa was applied in comparison with applying operating pressure 250 kPa the DU increased by 5% at 0.5 m and 1 m (Fig. 4c). Which, it is in agreement with^4^ who reported that the DU increased as the pressure increased, DU values were low with the riser 0.5 m for the pressures 62 and 82 kPa then it increased rapidly with the increase of the pressure. Moreover, in the tringle system, the DU increased by 2.42% in all the experiments when applying 200 kPa in compared with applying 250 kPa. Therefore, it is preferred to apply the 200 kPa in the square and the tringle systems, it is also does not consume high energy, that, it is in agreement with^56^ was applied two types of Floppy sprinkler system under various grades of height of riser and operating pressures.

**Figure 6:**
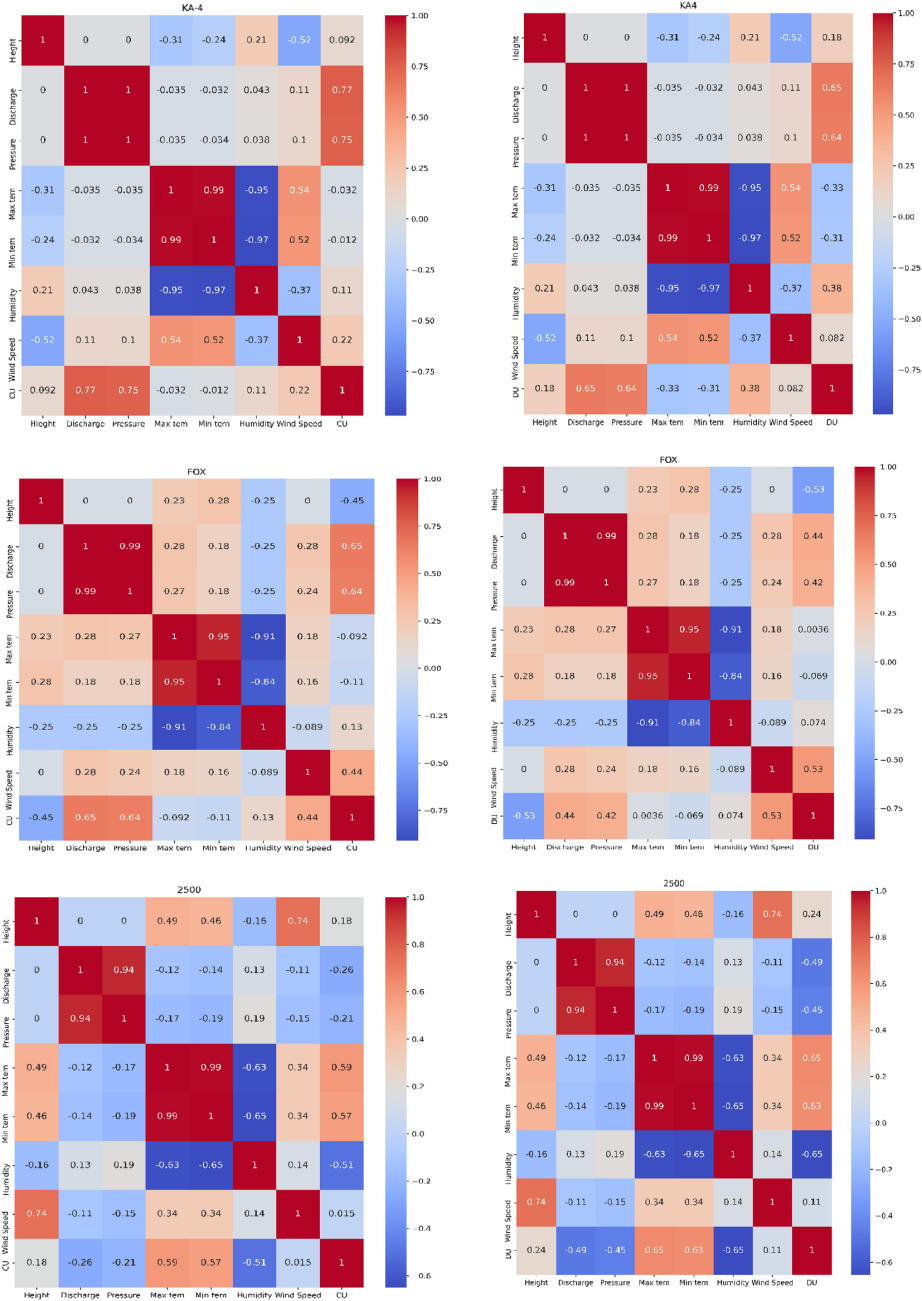
Correlation matrix between the hydraulic and climate variables and CU and DU.

Figure 6 showed the relationship between the CU and DU with other variables to figure out the positive and negative relationships. The CU was positive for height, discharge, pressure, relative humidity and wind speed, and it was the strongest positive relationship with the discharge by 0.77 and pressure by 0.75 for KA-4 sprinkler, however, the relationship was negative for maximum and minimum temperature due to the negative impact of temperature on the water distribution through the evaporation process.^57^ There was a negative correlation between DU and both wind speed and working pressure, although correlation coefficients were very low. There was a positive relationship between DU and the sprinkler height. However, the finding of^58^ concluded that the temperatures during the experiments ranged from (20.6 to 31.3 °C), which had an adverse effect on the uniformity of water distribution, when the temperature was increased, the uniformity of water distribution was decreased.

Moreover, for FOX sprinkler, the relationship was the same with the KA-4 however, the relation was 0.65 and 0.64 for discharge and pressure, respectively that were the strongest positive relationship (Fig. 6). On the other hand, for 2520 sprinkler, the relationship of CU was positive with height, wind speed, maximum and minimum temperature, although, the relationship was negative for discharge, pressure and relative humidity. On the other hand, the relationship of the DU with other variables were in agreement with the CU.

### Machine learning models

**Figure 7:**
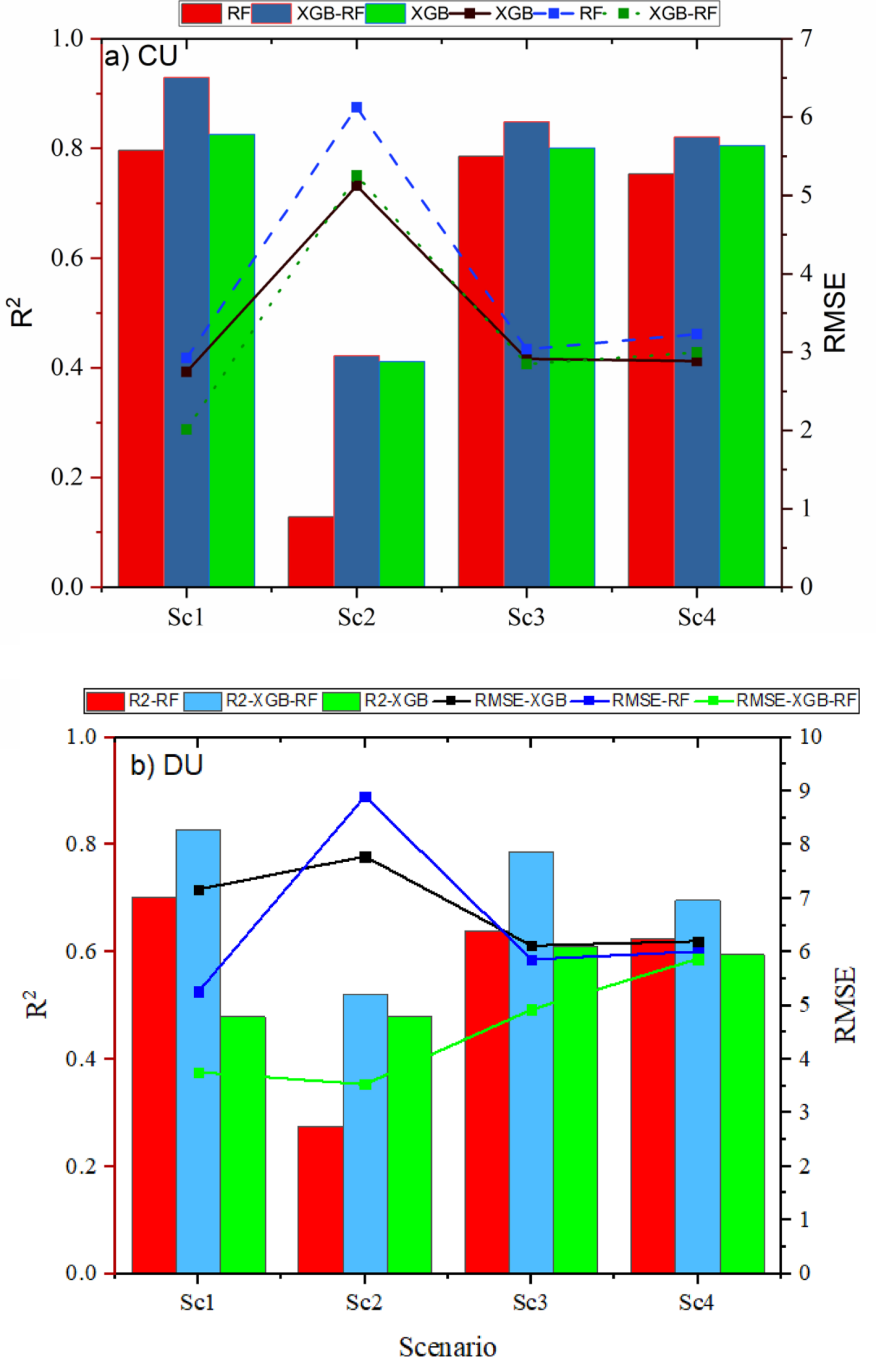
R^2^ and RMSE values of the applied models for CU and DU.

**Figure 8:**
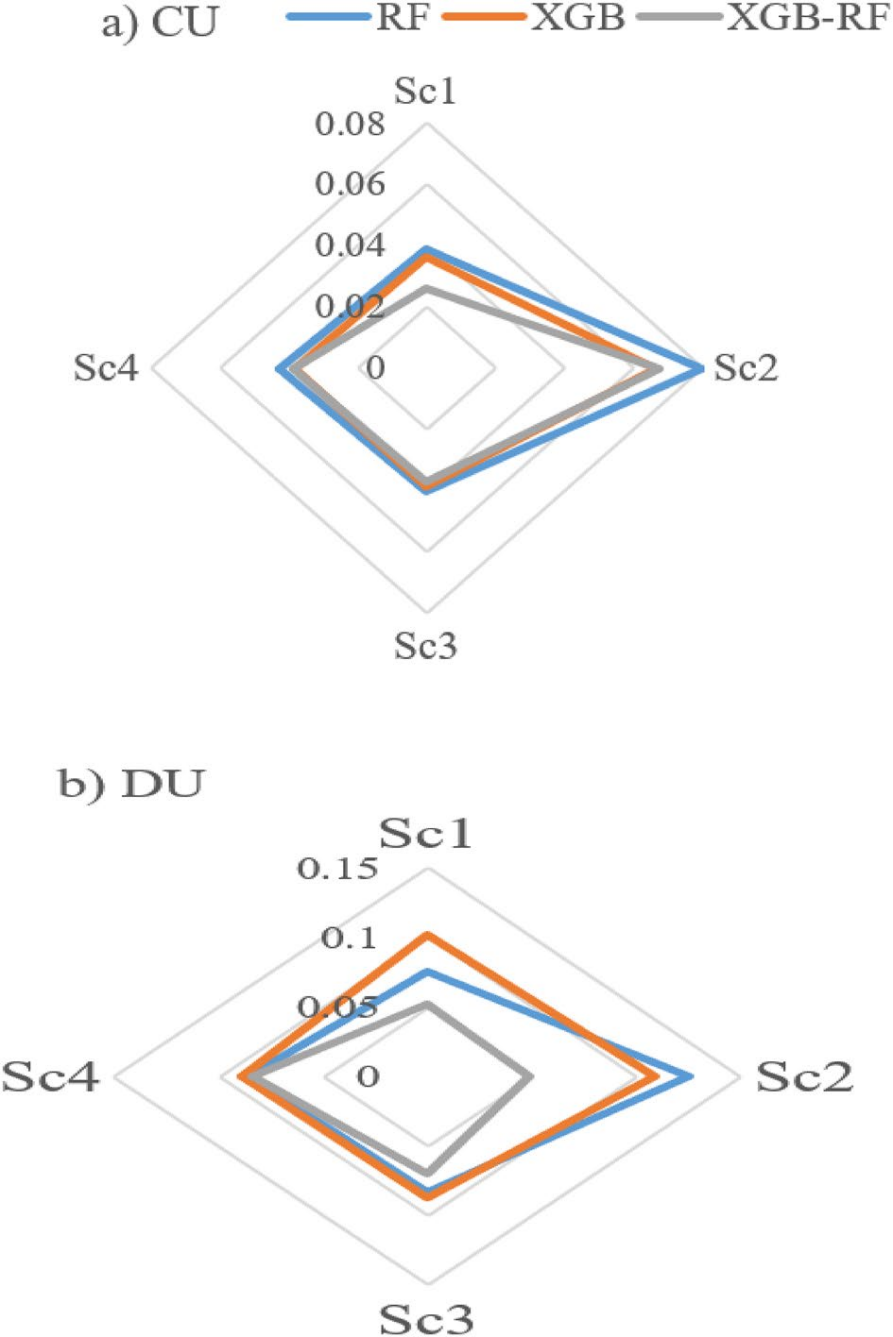
Radar chart of the SI index of the applied models.

**Figure 9:**
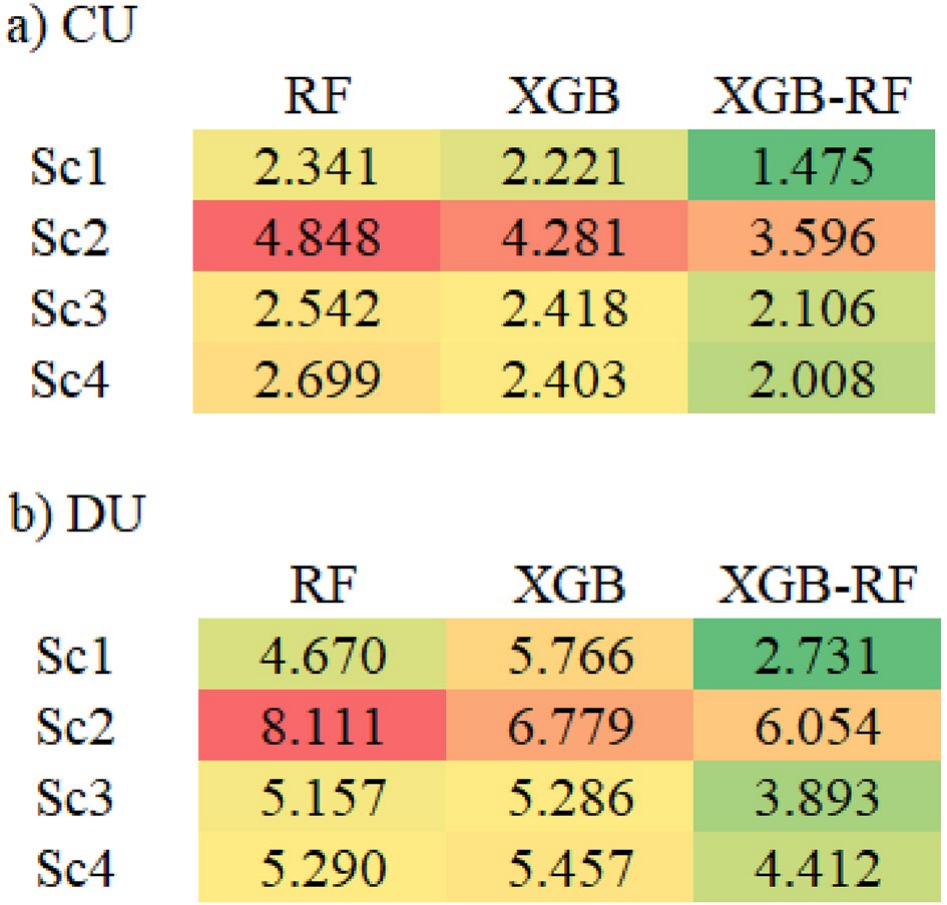
MAE index of the applied models.

Four scenarios were applied base on the hydraulic and climate variable to predict the water distribution uniformity based on machine learning algorithms (Table 2). In CU, the highest value of R^2^ was 0.80, 0.83 and 0.93 in RF, XGB and XGB-RF, respectively in the first scenario, however, the lowest value of R^2^ was 0.13, 0.41 and 0.42 in RF, XGB and XGB-RF, respectively in the second scenario. On the other hand, the lowest value of RMSE was 2.92, 2.75 and 2.01 in RF, XGB and XGB-RF, respectively in the first scenario, moreover, the highest value of RMSE was 6.12, 5.12 and 5.26 in RF, XGB and XGB-RF, respectively in the second scenario (Fig. 7a). On one hand, the lowest value of MAE was 2.34, 2.22 and 1.48 in RF, XGB and XGB-RF, respectively in the first scenario (Fig. 9a). In addition, the lowest value of SI was 0.04, 0.036 and 0.026 in RF, XGB and XGB-RF, respectively in the first scenario (Fig. 8a), therefore, based on the classification if the SI index, the XGB-RF model was classified as excellent for the first and second scenarios, which, the results are consistent with the results of^1^, where, were used five models (ANN, NF-GP, LS-SVM, NF-SC and GEP) to predict the distribution uniformity of water in the fixed sprinkler irrigation system, they achieved the best results for the value of R^2^ that was 0.970, 0.867 and 0.891 in ANN, NF-GP and LS-SVM, respectively, and SI index was 0.038, 0.080 and 0.072 in ANN, NF-GP and LS-SVM, respectively. The finding of^59^ was not in agreement with our results, where, their study demonstrated the effectiveness of using XGBoost as a sophisticated machine learning (ML) model to predict measurement phase levels over 5-min timescales with variety observation time windows.

The study of^60^, for the Random Forest model was developed using sample data derived from meteorological measurements including air temperature (Ta), relative humidity (RH), wind speed (WS) and photosynthetic active radiation (Par) to predict the lower baseline (T_wet_) and upper baseline (T_dry_) canopy temperatures for Chinese Brassica from 27 November to 31 December 2020 (E1) and from 25 May to 20 June 2021 (E2). The study showed the feasibility of using random forest model for prediction T_wet_ and T_dry_, by R^2^ was 0.90 and 0.91 in E1 and E2, respectively for T_wet_, also, the value of R^2^ is 0.88 and 0.89 in E1 and E2, respectively for T_dry_, which, it is in agreement with results this study when using RF to predict the distribution uniformity. The study of^61^ proposed a novel ET_0i_ estimation model named Particle Swarm Optimization (PSO)-XGBoost, as the main regression model and the parameters of XGBoost were optimized using the PSO algorithm to predict the ET_0i_ based on weather and soil moisture data of the two crops, the results indicated that the PSO algorithm could stably improve the parameters of the XGBoost model and able to precisely appreciation ET0i in a variety of data modes.

On contrast, the R^2^ decreased in the third scenario by 1.325%, 3.032% and 8.706% in comparison with the first scenario in RF, XGB and XGB-RF, respectively. However, the RMSE increased in the third scenario by 3.879%, 6.004% and 41.643%, respectively. The R^2^ increased by 16.66% in the first scenario when applied XGB-RF in comparison with RF. Moreover, The RMSE decreased by 31.25% in the first scenario when applied XGB-RF in comparison with RF. So, it is preferable to apply XGB-RF to predict the water distribution uniformity. Therefore, the first scenario is the best one, with the highest results which followed by the third scenario. On the other hand, the results were not significantly changed in scenario three and four, that means, the sprinkler height did not significantly impact on the CU and DU under the current conditions, maybe the reason is the impact of climate variables are not significantly during the experiment period. For example, wind speed was not high during the experiment period, thus, it did not impact on the water distribution uniformity. Further, for maximum and minimum temperature was not fluctuated seriously during the experiments beside all experiments were conducted in the morning and evening, that agree with the finding of^14^ who concluded that the effect of air losses that include drift and droplet evaporation was not highly significant impact on the water distribution uniformity, under the properly-operating sprinklers and low wind conditions, the water distribution uniformity decreased from 1 to 3%.

Therefore, the CU generally shows a tendency to decrease as wind speed increases. Since, it was not possible to compare the results of different parameters at a steady wind velocity, the comparisons were made using three different wind conditions, low (0–5 m/sec), moderate (5–7 m/sec) and high (> 7 m/sec) winds. The uniformity of water distribution was 90% at wind speeds ranging from 0 to 5 m/s, when wind speed become 5–7 m/s, it was 88% and at wind speed become greater than 7 m/s it was 75%^62^. Moazed et al.^63^ studying the wind speed effect on distribution uniformity, found a CU of 89% for low wind speed and 67% for high wind speed conditions. According to^64^, both water depth intensity and wind direction affects the destination of particles. Therefore, the wind has a considerable influence on the area’s water distribution. So, to promote an increase in water distribution uniformity, it is necessary to perform the irrigations during nighttime, when wind speed has its lowest values. Overnight, the irrigation will also have a higher application efficiency due to reduced rates of evaporation and drift of water droplets^58^. When winds were lower than 2 m/s, the mean CU value was 87.4%. For wind speeds ranging from 2 to 4 m/s, the average CU value was 85.3%; and for wind speed higher than 4 m/s, the average CU value was 77.2%. Ten evaluations with variable winds were available. Five of them had CU values higher than 90%, in three others CU values were over 87%, and the other two had CU values higher than 75%, but with wind speeds higher than 4.3 m/s. This shows the positive effect of wind variations (either in speed or direction) on uniformity. Uniformity only increased as a function of pressure with low winds^57^. Reduced wind speed values are vital to ensure an adequate irrigation efficiency, especially for sprinkler systems, since such variable might result in major impacts on water application and distribution^18,19^. A strategy to overcome such problem is the practice of overnight irrigations since it might be a way to reduce the effects of high wind speed^58^. According to^65^, night periods have lower thermal gradients, resulting in reduced wind speed.

On contrast, for the DU, the lowest value of R^2^ is 0.275, 0.481 and 0.522 in RF, XGB and XGB-RF, respectively in the second scenario, on the other hand, the highest value of R^2^ is 0.701, 0.479 and 0.827 in RF, XGB and XGB-RF, respectively in the first scenario (Fig. 7b). Similar conclusions were studied by^66^ used XGBoost regression (XGBR) model to calculate ET, his study was for 3 years (2019–2021) to model the effects on ET of eight meteorological factors [net solar radiation (Rn), mean temperature (Ta), minimum temperature (Tamin), maximum temperature (Tmax), relative humidity (RH), minimum relative humidity (RHmin), maximum relative humidity (RH max) and wind speed (V)] using a greenhouse drip irrigated tomato crop ET prediction model (XGBR-ET) that was based on XGBoost regression (XGBR). The model was compared with seven other common regression models. Were lined up, the eight models in terms of forecast precision, XGBR-ET > GBR-ET > SVR-ET > ABR-ET > BR-ET > LR-ET > KNR-ET > RFR-ET. The parameters of the XGBR-ET model were ablated to show that the order of importance of meteorological factors on XGBR-ET was Rn > RH > RHmin > Tmax > RH max > Tamin > Ta > V. The values of performance evaluation R^2^, RMSE and MAE are 0.981, 0.163 and 0.132, respectively.

However, for the DU, the highest value of SI is 0.12 and 0.11 in RF and XGB, respectively in the second scenario which is classified as a good model (Fig. 8b), but for XGB-RF is 0.083 in the fourth scenario. In addition, the lowest value of SI is 0.07 and 0.05 in RF and XGB-RF, respectively in the first scenario (Excellent model), while is 0.08 in the third scenario in XGB model which is classified as an excellent model (Fig. 8b), therefore, the model is classified as excellent. Moreover, the lowest value of MAE is 4.6, 5.7 and 2.7 in RF,

XGB and XGB-RF, respectively, in the first scenario (Fig. 9b), also, the highest value of MAE is 8.11, 6.77 and 6.05 in RF, XGB and XGB-RF, respectively, in the second scenario (Fig. 9b) for the DU.

## Conclusion

This study aimed to study the evaluation of the water distribution uniformity under sprinkler irrigation system-based on hydraulics (pressure, discharge, height) and climate variables (maximum temperature, minimum temperature, humidity and wind speed). This study compared three sprinklers’ types to obtain the highest possible distribution uniformity with the lowest energy. Based on multicriteria, it was found that the highest CU value was 86.7% in the square system and 87.3% in the triangular system of the sprinkler 2520. By applying 150, 200 and 250 kPa, it is preferable to use the triangle system applied by 200 kPa. Further, in the triangular system, the CU values at 200 kPa are larger than the square system, so, it is preferable to use the triangle system. For the correlation between the CU and climate variables, the strongest positive relationship was found for maximum and minimum temperature for 2520 sprinkler.

Four machine learning scenarios were applied to predict the water distribution uniformity based on single and hybrid machine learning algorithms. For CU and DU, the XGB-RF model was the best model all scenarios and the first scenario was the best scenario. The R^2^ increased by 16.66% in the first scenario when applied XGB-RF in comparison with RF. Moreover, The RMSE decreased by 31.25% in the first scenario when applied XGB-RF in comparison with RF. So, it is preferable to apply XGB-RF to predict the water distribution uniformity. Therefore, the first scenario is the best one, with the highest results followed by the third scenario.

## Data Availability

The datasets generated and/or analyzed of the current study are available from the crossponding author on reasonable request.
